# CALM1 promotes progression and dampens chemosensitivity to EGFR inhibitor in esophageal squamous cell carcinoma

**DOI:** 10.1186/s12935-021-01801-6

**Published:** 2021-02-18

**Authors:** Tao Liu, Xiujuan Han, Shutao Zheng, Qing Liu, Aerziguli Tuerxun, Qiqi Zhang, Lifei Yang, Xiaomei Lu

**Affiliations:** 1 State Key Laboratory of Pathogenesis, Prevention and Treatment of High Incidence Disease in Central Asia, The First Affiliated Hospital of Xinjiang Medical University, Xinjiang Uygur Autonomous Region, Urumqi, PR China; 2 Department of Clinical Laboratory, The First Affiliated Hospital of Xinjiang Medical University, Xinjiang Uygur Autonomous Region, Urumqi, PR China

**Keywords:** Esophageal squamous cell carcinoma (ESCC), CALM1, EGFR, Afatinib, Chemosensitivity, Cancer progression

## Abstract

**Background:**

Calmodulin1 (CALM1) has been identified as one of the overexpression genes in a variety of cancers and EGFR inhibitor have been widely used in clinical treatment but it is unknown whether CALM1 and epidermal growth factor receptor (EGFR) have a synergistic effect in esophageal squamous cell carcinoma (ESCC). The aim of the present study was to explore the synergistic effects of knock-out CALM1 combined with EGFR inhibitor (Afatinib) and to elucidate the role of CALM1 in sensitizing the resistance to Afatinib in ESCC.

**Method:**

Immunohistochemistry (IHC) and qRT-PCR were used to examine the expression of CALM1 and EGFR in ESCC tissues. Kaplan–Meier survival analysis was used to analyze the clinical and prognostic significance of CALM1 and EGFR expression in ESCC. Furthermore, to evaluate the biological function of CALM1 in ESCC, the latest gene editing technique CRISPR/Cas9(Clustered regularly interspaced short palindromic repeats)was applied to knockout CALM1 in ESCC cell lines KYSE150, Eca109 and TE-1. MTT, flow cytometry, Transwell migration, scratch wound-healing and colony formation assays were performed to assay the combined effect of knock-out CALM1 and EGFR inhibitor on ESCC cell proliferation and migration. In addition, nude mice xenograft model was used to observe the synergistic inhibition of knock-out CALM1 and Afatinib.

**Results:**

Both CALM1 and EGFR were found to be significantly over-expressed in ESCC compared with paired normal control. Over-expressed CALM1 and EGFR were significantly associated with clinical stage, T classification and poor overall prognosis, respectively. In vitro, the combined effect of knock-out CALM1 mediated by the lentivirus and EGFR inhibitor was shown to be capable of inhibiting the proliferation, inducing cell cycle arrest at G1/S stage and increasing apoptosis of KYSE-150 and Eca109 cells; invasion and migration were also suppressed. In vivo, the results of tumor weight and total fluorescence were markedly reduced compared with the sgCtrl-infected group and sgCAML1 group.

**Conclusion:**

Our data demonstrated that knock-out of CALM1 could sensitize ESCC cells to EGFR inhibitor, and it may exert oncogenic role via promotion of EMT. Taken together, CALM1 may be a tempting target to overcome Afatinib resistance.

## Introduction

Esophageal cancer (EC) is one of the most common gastrointestinal tract malignancies and ranks as the sixth most important cause of cancer mortalities globally, with an incidence of estimated 509,000 new deaths every year [1, 2]. As one of the most common pathohistological subtypes of ESCC, ESCC usually composes over 90% of all EC cases in areas of Asia and Sub-Saharan Africa [1, 3]. Despite the advances in diagnosis and treatment, ESCC still carries a poor prognosis [4], and the 5-year overall survival rate ranges from 15 to 25%[5]

CALM is a ubiquitous calcium ion (Ca^2+^) receptor protein, mediating a large number of signaling processes; it is highly conserved from an evolutionary standpoint [6–10]. CALM with a sequence of 148 amino acids is present in all eukaryotic cells [11]. In humans, CALM is encoded by three different genes (CALM1, CALM2, CALM3), each of which has unique selective regulation, tissue specificity, and alternative splicing, but surprisingly, they all produce same protein [10, 12]. However, although specific cells can express these three genes, they do not necessarily all have the same functional roles because the three transcripts can be differentially processed by post-transcriptional regulation or subcellular distribution [13]. In this study we focus on CALM1. CALM1 is composed of Ca^2+^-binding EF-hands, and participates in signaling pathways that modulates proliferation, motility and differentiation [14]. Several studies found that the expression level of CALM1 was markedly associates with many kinds of cancer, including bladder cancer [15], prostate cancer [16] and nasopharyngeal carcinoma [17]. As far as CALM is concerned, numerous investigations have been carried out on mechanistic aspects, mainly in the cell proliferation, programmed cell death and autophagy. CALM/Ca^2+^ binding to the SH2 domains of the p85 subunit of PI3Kα stimulates PI3Kα/Akt/mTOR signaling, and thereby regulating cell proliferation and growth [18, 19]. CALM also regulated EGFR’s tyrosine kinase activity [20] which activates Ras and PI3Kα and has essential roles in programmed cell death and autophagy [18]. However, the biological function of CALM1 and its regulatory mechanism in ESCC are rarely studied.

Compared with tissues of healthy population, the mRNA and protein EGFR expression are significantly elevated, which can be applied as a meaningful marker in early diagnosis, and judgment of prognosis of ESCC [21]. After combined with and activated EGFR by EGF, which can initiate a series of cellular reactions, including cell proliferation, as well as resistance to apoptosis, invasion, and metastasis and neovascularization [22, 23]. The EGFR gene encodes a membrane glycoprotein responsible for the upregulation of EGFR signaling. The success of EGFR tyrosine kinase inhibitor (TKIs) have provided a powerful validation for precision cancer medicine because the over-expression and mutations on EGFR plays an important carcinogenic role in a variety of solid tumors such as head and neck, breast, lung, and colorectal cancer, and numerous EGFR inhibitor have been widely used in clinical treatment [24–27]. Fumiyuki Sato et al. [28] reported that EGFR inhibitor prevent induction of cancer stem like cells in ESCC by suppressing EMT. In view of these previous findings, we hypothesized that CALM1 and EGFR may play a synergistic role in the development of ESCC. However, up to now, the relationship of CALM1 and EGFR in the progression of ESCC remains unknown. Herein, we undertake the study to present our results of characterization of CALM1 and EGFR and to analyze its clinical relevance in ESCC.

## Methods

### Cell culture

Two human ESCC cell lines, KYSE150 and TE-1, were obtained from the Chinese Academy of Sciences (Shanghai, China), and Eca109 cells were from Wuhan University(Wuhan, China).The three cell lines were cultured in RPMI-1640 (Invitrogen, Life Technologies, Carlsbad, CA, USA) supplemented with 10% fetal bovine serum (Gibco, Carlsbad, CA, USA) and 1% penicillin–streptomycin (Gibco;Thermo Fisher Scientifc, Inc.). All the ESCC cell lines were cultured in a 5% CO^2^ humidified incubator at 37 °C.

### Tissue microarray

Tissue microarrays of clinical samples consisted of ESCC and paired normal adjacent tissues (NAT).One tissue microarray included 34 paired cases of ESCC and matched NAT (catalog number: # HEsoS180Su08; Outdo Biotech, Shanghai, China), and another 50 additional independent, subjected to esophagectomy, obtained from the First Affiliated Hospital of Xinjiang Medical University. Tumor tissues and clinicopathological parameters were collected after obtaining the informed consent from each participant involved. The study was approved by Medical Ethics Committee of the First Affiliated Hospital of Xinjiang Medical University (Approval Number:2018K06–20).

### Immunohistochemistry (IHC)

Tissue microarrays were de-waxed and hydrated, boiled in 0.01 M citrate buffer, and treated with 3% hydrogen peroxide after natural cooling. The primary polyclonal rabbit anti CALM1(catalog number: #10,541–1-AP; dilution at 1:400; Proteintech, Wuhan, China), EGFR (catalog number: # 18,986–1-AP;1:600; Proteintech), cleaved caspase-3(Catalog number: 19677–1-AP, dilution at 1:200; Proteintech, Wuhan, China) and Ki-67(Catalog number: 27309–1-AP, dilution at 1:10,000; Proteintech, Wuhan, China) were incubated overnight in 4 °C by adding drop of glass slide, followed by treatment with biotinylated anti-rabbit secondary antibody (Catalog number:# ZLI‐9032, Zhongshan Jinqiao Biotechnology) for 60 min at 37 °C overnight in 4 °C. The immunostainings results were evaluated by two pathologists (Qing Liu and Xiaomei Lu) under optical microscopy and cellular sub-localization of immunostaining was assessed in each section. The intensity of staining was divided into four grades (0, none; 1, weak; 2, moderate; and 3, strong) and percentage of positive cells (0, < 10%; 1, 10%-25%; 2, 25%–50%; and 3, > 50%). According to the immunoscoring (staining intensity plus positive cell score), ESCC patients were divided into two groups, specifically "low expression "(total score,0–3) and" high expression "(total score,4–6), which were used to analyze the prognostic significance of EGFR and CALM1 in ESCC.

### CRISPR-Cas9 knock-out construction and lentiviral transfection

The LentiCRISPR P2A-GFP-CALM1 CRISPR/Cas9 construct was outsourced to Shanghai Genechem Co Ltd (Shanghai, China). Lentiviruses carrying green fluorescent protein (GFP) is along with scrambled Lv-sgRNA-control (sgCtrl) and CALM1 sgRNA (Lv-sgCALM1-1, Lv-sgCALM1-2, and Lv-sgCALM1-3). A suitable amount of lentivirus was added to the culture medium of ESCC for transduction, according to the multiplicity of infection (MOI), and the cells were incubated further for 8 h. After 72 h, all fluorescent cells were sorted via flow cytometry and transfection efficiency was evaluated by Western blots and Quantitative real-time polymerase chain reaction (qRT-PCR). The sgRNA target sequences we used were as follows: sgCALM1-1(GACGGACAAGTCAACTATGAA), sgCALM1-2 (CGTGAGGCATTCCGAGTCTTT), sgCALM1-3(AGAAGCTGAATTGCAGGATAT), and sgRNA control (TTCTCCGAACGTGTCACGT).

### Quantitative real-time polymerase chain reaction (qRT-PCR)

Total RNA was extracted with TRIzol reagent and then reversely transcribed into cDNA using a Pria Revert Aid First Strand cDNA Synthesis Kit (catalog number: #A5001, promega). Following the manufacturer’s protocols, Real-time PCR was performed using a SYBR Green Premix PCR Master Mix (catalog number: #DRR041B, TAKARA). Relative mRNA expression of CALM1 and EGFR was calculated using the 2^−ΔΔCt^ method after being normalized to GAPDH, which served as internal loading control. PCR was performed with the following primer sets: CALM1 forward, 5′-GGTCAGAACCCAACAGAA-3′ and reverse, 5′-AGACTCGGAATGCCTCA-3′; and EGFR forward, 5′-AGGCACGAGTAACAAGCTCAC-3′ and reverse, 5′-ATGAGGACATAACCAGCCACC-3′. GAPDH forward, 5′-TGACTTCAACAGCGACACCCA-3′ and reverse, 5′-CACCCTGTTGCTGTAGCCAAA-3′. All experiments were performed independently three times and shown was the representative one.

### Western blots

The cells were lysed on ice with the RIPA (Radio Immunoprecipitation Assay) lysis buffer (Thermo Fisher Scientific, USA) for 30 min to prepare the cell suspension, followed by centrifugation at 14,000 rpm for 10 min at 4 °C. The protein concentration was determined with bicinchoninic acid protein assay (Thermo Fisher Scientific, USA), 0.1 mg total protein were subjected to 10% SDS-PAGE separation and then transblotted to PVDF membranes (Millipore, Billerica, MA, USA). The membrane was blocked in 5% nonfat milk for 1 h at room temperature, and then incubated with the primary antibodies. Target proteins were detected by using specific antibody against CALM1 (catalog number: #10,541–1-AP; dilution at 1:800; Proteintech Group, Wuhan, China). GAPDH (catalog number: 10494–1-AP; dilution at1:5000, Proteintech Group, Wuhan, China) was chosen as an internal control and the CALM1 and GAPDH dilutions were incubated at 4 °C with gentle shaking overnight. Then, secondary antibody (goat anti-rabbit, catalog number:SA00002-2, Proteintech Group, Wuhan, China) were added onto the membrane for incubation at room temperature for 2 h.The blots were visualized with Pierce™ ECL Western Blotting Substrate (Thermo Fisher Scientific, CA, USA), according to the manufacturer's protocol.

### MTT assay

Cells were placed into the 96-well plates at the density of 4 × 10^4^/mL in RPMI-1640. At the designated time point, the cells were coated with 100 μL sterile MTT (Sigma-Aldrich) in an incubator with 5% CO_2_ for 4 h at 37 °C. Afatinib was added with the desired drug treatment concentrations ranging from 0 to 20 μM and incubated for 72 h. The reaction waster was performed by removing the culture medium and then adding 100 μL of dimethyl sulfoxide (Sigma-Aldrich) for 0.5 h to dissolve the formaldehyde. Finally, absorbance values were measured at 490 nm. The IC_50_ (half-maximum inhibitory concentration) was used as the measure of relative cytotoxicity.

### Apoptosis assay and cell cycle

After transfection with sgCtrl and sgCAML1-1 with or without EGFR inhibitor for 72 h, ESCC were collected after washing twice with PBS. For cell cycle, cells were fixed in 70% ice-cold ethanol overnight, then washed twice with PBS and stained with 10 μg/mL RNase A in the dark for 15 min at room temperature. The analysis was performed by a flow cytometer (BD FACS Calibur; BD Biosciences, Brea, CA, USA). For analysis of apoptotic cells, it was analyzed by flow cytometry using an FITC Annexin V Apoptosis Detection Kit (Thermo Fisher Scientific) according to the manufacturer’s instructions after harvesting cells.

### Transwell assay

Cells were added in the upper well of a Transwell chamber (8 μm pore size) that was pre–coated with 50 µL Matrigel (BD, Bedford, MA). Cells at a density of 1 × 10^5^ cells per well were placed into the upper chambers in 600 μL serum-free RPMI 1640 containing 10% FBS, and the cells were incubated at 37 °C in a CO2 incubator(5% CO2). After 24 h, non–invaded cells on the upper chamber of the filter were scraped off with swabs. The migrated cells on the lower chamber were fixed with 4% formaldehyde for 10 min, and stained with 0.1% crystal violet for 15 min. Finally, the number of invaded cells were photographed under a light microscope and counted using Image J software (NIH, Bethesda, MA, USA).

### Wound healing assay

ESCC cells (5 × 10^4^) were seeded in 6-well plates to reach more than 90% confluence. The samples were then scratched manually using a pipette tip. After scratching with a sterile pipette tip, removal of cell debris by washing 3 times with PBS, the wounded cell samples were then cultured in serum-free medium. Images were acquired at 0 h, 4 h, 8 h and 24 h post scratching by using a microscope. Four wound areas were photographed on each plate and counted under an Olympus inverted fluorescence phase-contrast microscope (Tokyo, Japan). Then the percentage of wound closure was calculated by the following formula: wound closure (%) = (original gap distance-gap distance at the indicated time)/original gap distance × 100%. wound healing assay was performed independently three times, with each group assayed in triplicate.

### Colony formation assay

KYSE150 and Eca109 cells transfected with sgCtrl, sgCALM1-1 were plated in 6-well plates (1000 cells/well) and incubated at 37 °C for 14 days to allow colony formation. In the drug treatment group, the medium was changed with fresh medium containing Afatinib or vehicle (DMSO) every 2 days. The cell medium was subsequently removed. Cells were washed using PBS and fixed with 4% paraformaldehyde for 10 min at room temperature. The cells were stained with crystal violet kit (Beyotime Institute of Biotechnology, Shanghai, China) for 15 min at room temperature. The colonies were washed, photographed by camera and counted using ImageJ software. Colony formation experiment was performed independently three times, with each group assayed in triplicate.

### Tumorigenesis in nude mice and in vivo imaging

Nude mice (4 weeks old) were purchased from Shanghai Lingchang Biological Technology Co., Ltd. All animals (22 ± 1.5 g) were handled according to the Guide for the Care and Use of Laboratory Animals and were housed at a controlled temperature (22–28˚C) and humidity (50%) under a 12-h light/dark cycle. All mice were randomly divided into three groups: sgCtrl group, sgCAML1-1 group, sgCAML1-1 plus EGFR inhibitor group. Then, the stably cells (4 × 10^6^ for each side) were suspended in PBS and implanted subcutaneously into male BALB/c nude mice. Animals in the sgCAML1-1plus EGFR inhibitor group treated with 20 mg/kg paclitaxel every 3 days once when tumor size reached about 100 mm^3^ intraperitoneally (i.p.). After 7 days, the tumor weight was measured every 3 days for 4 weeks. After 27 days of monitoring, in vivo imaging of animals before they are sacrificed and the tumors were dissected and weighted.

### Statistical analysis

Data were expressed as the mean ± standard deviation (SD), using SPSS for Windows version 19.0 (SPSS, Inc., Chicago, IL, USA). Student t test or one-way analysis of variance (ANOVA) was used to evaluate the differences among groups, and chi-square and Fisher's exact tests were applied to analyze correlation between CALM1/EGFR expression and clinicopathological characteristics. The Kaplan–Meier survival curve and log rank test were used to plot the survival curves and estimate survival rates. A two-tailed P < 0.05 was taken as significant in all tests.

## Results

### High CALM1 and EGFR expression were significantly associated with metastasis and poor prognosis in ESCC

**Fig. 1:**
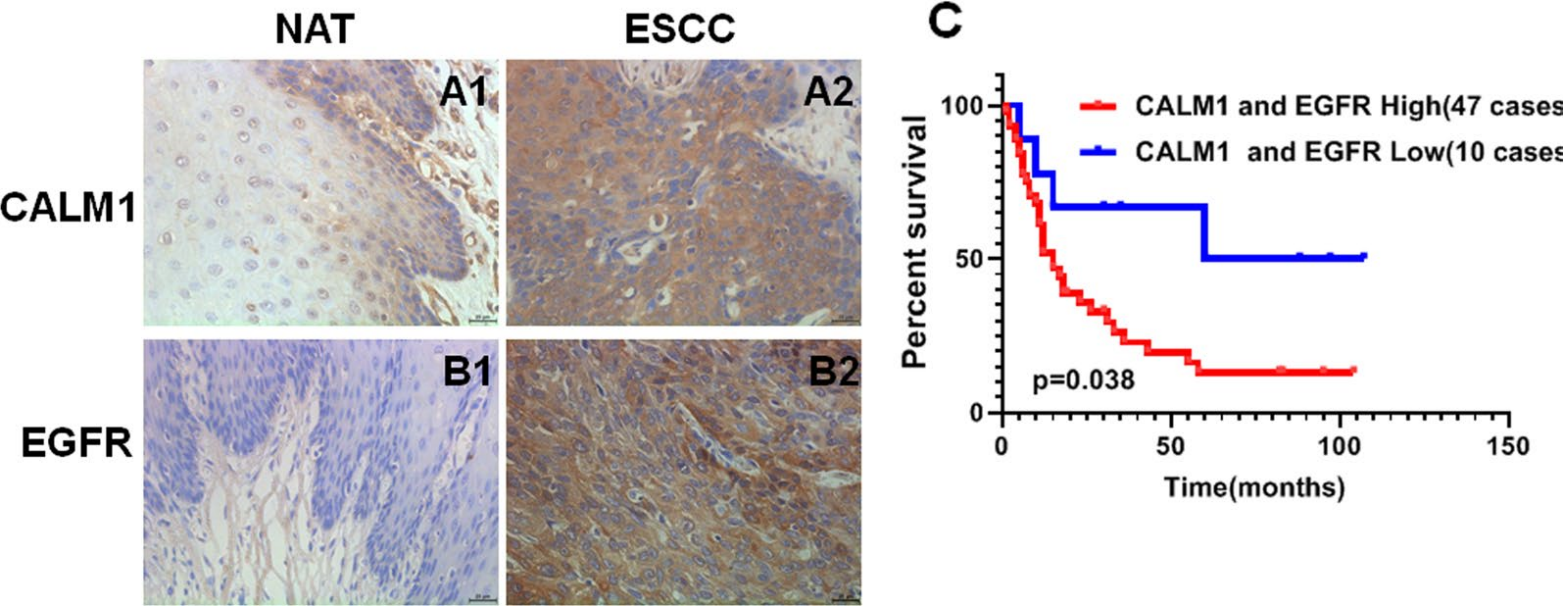
High CALM1 and high EGFR expression in ESCC was significantly correlated with metastasis and poor clinical prognosis. A1, A2, Immunostaining of CALM1 in ESCC and paired normal control. B1, B2, Immunostaining of EGFR in ESCC and paired normal control. The scale bar represents 25 μm. Magnification fold is 400 × . C, Kaplan‐Meier overall survival curves for all 84 patients with ESCC stratified by high and low expression of CALM1 and EGFR (*NAT* normal adjacent tissues)

To understand the pathological significance of CALM1 and EGFR expression, we first detected the expression levels of CALM1 and EGFR in 84 paraffin-embedded human ESCC and paired NAT tissue blocks, by immunohistochemistry (IHC). IHC analysis revealed that various ESCC tissues show higher expression of CALM1 and EGFR in ESCC tissues compared to the NAT tissues (p < 0.001, Fig. 1a, Table 1). The correlation between the levels of these two proteins in the ESCC tissue and the clinicopathological parameters of the 84 ESCC patients was analyzed and presented in Table 1. Expression of CALM1 and EGFR was not found to be correlated with gender, age, Tumor diameter (cm), T classification but closely related to clinical stage (Table 1). Kaplan–Meier survival analyses revealed a significantly shorter overall survival time for patients with high CALM1 and EGFR expression relative to patients with low CALM1 and EGFR expression (p < 0.05, Fig. 1c). Notably, the CALM1 expression was positively correlated with EGFR in clinical tissues of ESCC (p < 0.001, Fig. 1b, Table 2).

**Table 1:** Correlation between CALM1 and EGFR expression and clinicopathologic characteristics in 84 cases of ESCC

Characteristics	CALM1 expression		χ2	P Value	EGFR expression		χ2	P Value
	High	Low			High	Low		
Type								
ESCC	68	16	27.327	0.000	65	19	26.565	0.000
NAT	35	49			32	52		
Gender								
Male	50	11	0.149	0.758	50	11	2.677	0.102
Female	18	5			15	8		
Age (y)								
≤ 60	25	5	0.172	0.778	23	7	0.014	0.556
> 60	43	11			42	12		
Tumor diameter (cm)								
< 5	46	12	0.328	0.766	45	13	0.005	1.000
≥ 5	22	4			20	6		
Clinical stage								
I	19	11	9.395	0.004	18	12	8.005	0.007
II – III	49	5			47	7		
T classification								
T1 – T2	23	7	0.556	0.564	22	8	0.437	0.345
T3 –T4	45	9			43	11		
Lymph node metastasis								
N0	46	9	0.744	0.397	41	14	0.732	0.584
N1–N3	22	7			24	5

**Table2:** Relationship between CALM1 and EGFR expression in patients with ESCC

CALM1 expression	EGFR expression		Contingency coefficient	P Value
	Positive	Negative		
Positive	59	9	17.960	0.000
Negative	6	10		

### Knockout of CALM1 and treated with EGFR inhibitor markedly impaired the proliferation, cell cycle and increased apoptosis of ESCC cells

**Fig. 2:**
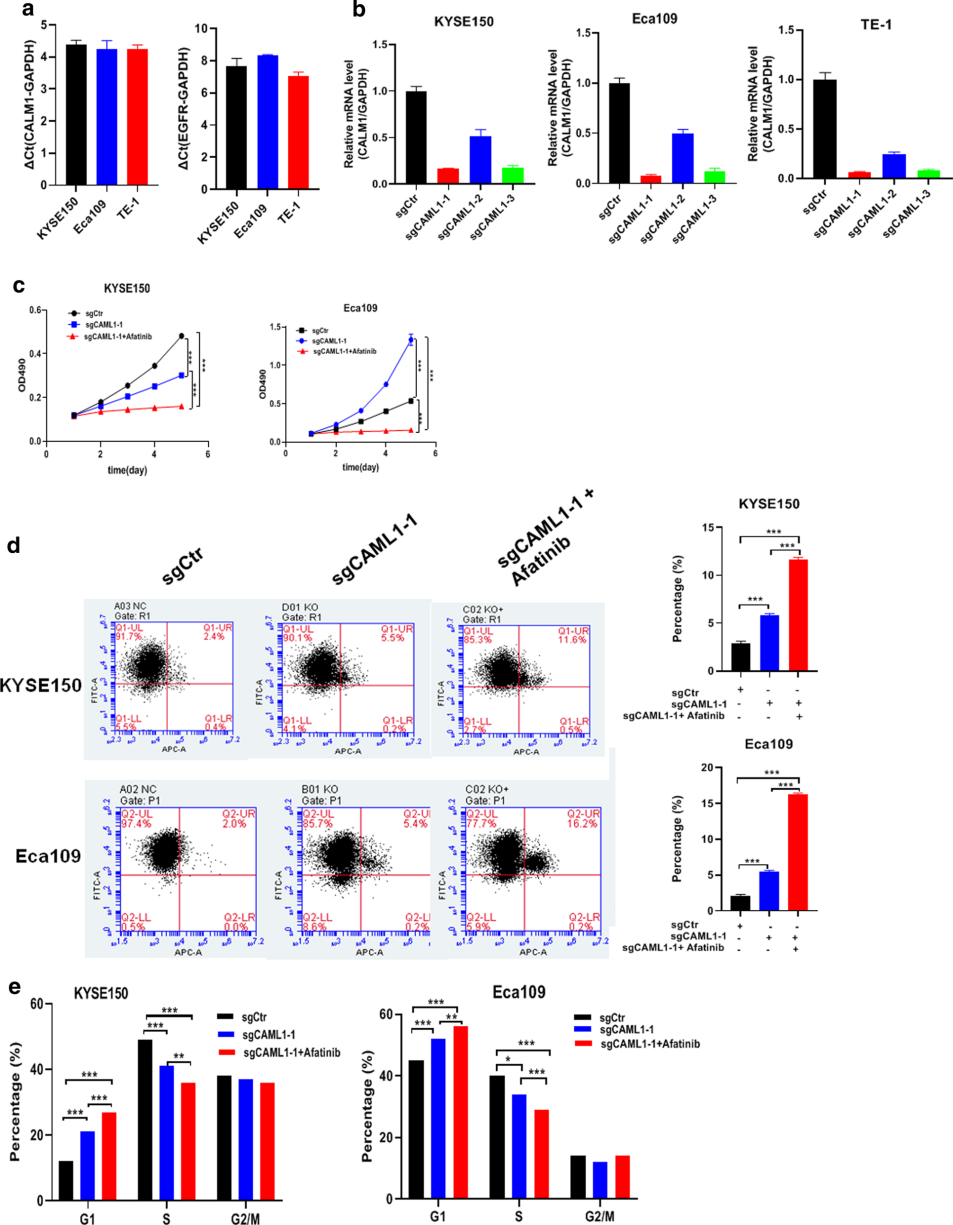
Knockout of CALM1 and treated with EGFR inhibitor markedly impairs the proliferation, apoptosis and cell cycle of ESCC cells. **a** Basal expression of CALM1 and EGFR on mRNA by qRT-PCR was detected in three tested ESCC cell lines. **b** qRT-PCR showing remarkable silencing efficiency in three ESCC cell lines infected with sgCALM1-1, sgCALM1-2 and sgCALM1-3. **c** Comparison of the proliferation of ESCC cell lines after knockout of CALM1 with or without inhibition of EGFR by MTT. Cells were pretreated with the inhibitors for 1 h and maintained in culture. **d** Influence of CALM1 and EGFR inhibitor on apoptosis in ESCC cells, analyzed by using flow cytometry. The percentage of Annexin V-FITC-positive cells to the total cells is shown in bar graphs. **e** Flow cytometry analysis of the effects of EGFR inhibitor treatment on cell cycle in ESCC after CALM1 knockout. All assays were performed in triplicate and the results are presented as the mean ± S.D. in all panels (**c**, **d**, **e**). CALM1, calmodulin1; EGFR, epidermal growth factor receptor. ESCC, esophageal squamous cell carcinoma; MTT, 3-(4, 5-dimethyl-2-thiazolyl)-2,5-diphenyl-2H-tetrazolium bromide; sgRNA, guide RNA. *P < 0.05, **p < 0.01, ***p < 0.001(Student's t-test)

Having understood the clinicopathological significance of the CALM1 and EGFR in vivo in ESCC, therefore we hypothesized that knockout of CALM1 and treatment with EGFR inhibitor (Afatinib) could markedly impair the proliferation and apoptosis in vitro in ESCC cell lines. To test the hypothesis, firstly, the basal level of CALM1 and EGFR on mRNA was evaluated using qRT-PCR, in a panel of human ESCC cell lines—KYSE150, Eca109 and TE-1. Results showed these three ESCC cell lines, the basal level of CALM1 and EGFR was higher in KYSE150 and Eca109 cell lines than that in TE-1 cells lines (Fig. 2a). On the basis, KYSE150 and Eca109 cell lines were selected as cell model to further investigate the biological roles of CALM1 and EGFR in ESCC cells. qRT-PCR data showed that sgCALM1-1 successfully achieved significant depletion of CALM1 in these two cell lines (Fig. 2b). To investigate the effect that synergistic reaction of CALM1 and EGFR exerted over proliferation and apoptotic variation of ESCC cells, we carried out MTT assay and flow cytometry after KYSE150 and Eca109 cell lines were transfected with lentiviral-based knockdown of CALM1. The IC50 value of Afatinib for KYSE150 and Eca109 cells were 8.80 μM and 4.01 μM, respectively (Additional file 1: Fig S1). It was exhibited that depletion of CALM1 moderately inhibited the proliferation (p < 0.001, Fig. 2c) and increased apoptotic (p < 0.001, Fig. 2d). More important, treatment with Afatinib can markedly slow down the proliferation (p < 0.001, Fig. 2c) and increase apoptotic (p < 0.001, Fig. 2d), compared with control and sgCALM1-1 group, strongly suggesting the tumor-promoting role of CALM1 and EGFR in ESCC cells. Cell cycle analysis revealed that KYSE150 and Eca109 cells with CALM1 knockout arrested in G1 and S phase after EGFR inhibitors treatment than the sgCtrl group and sgCALM1-1 group (p < 0.001, Fig. 2e). These findings indicated that reducing CALM1 and EGFR expression inhibits the G1/S phase transition.

**Fig. 3:**
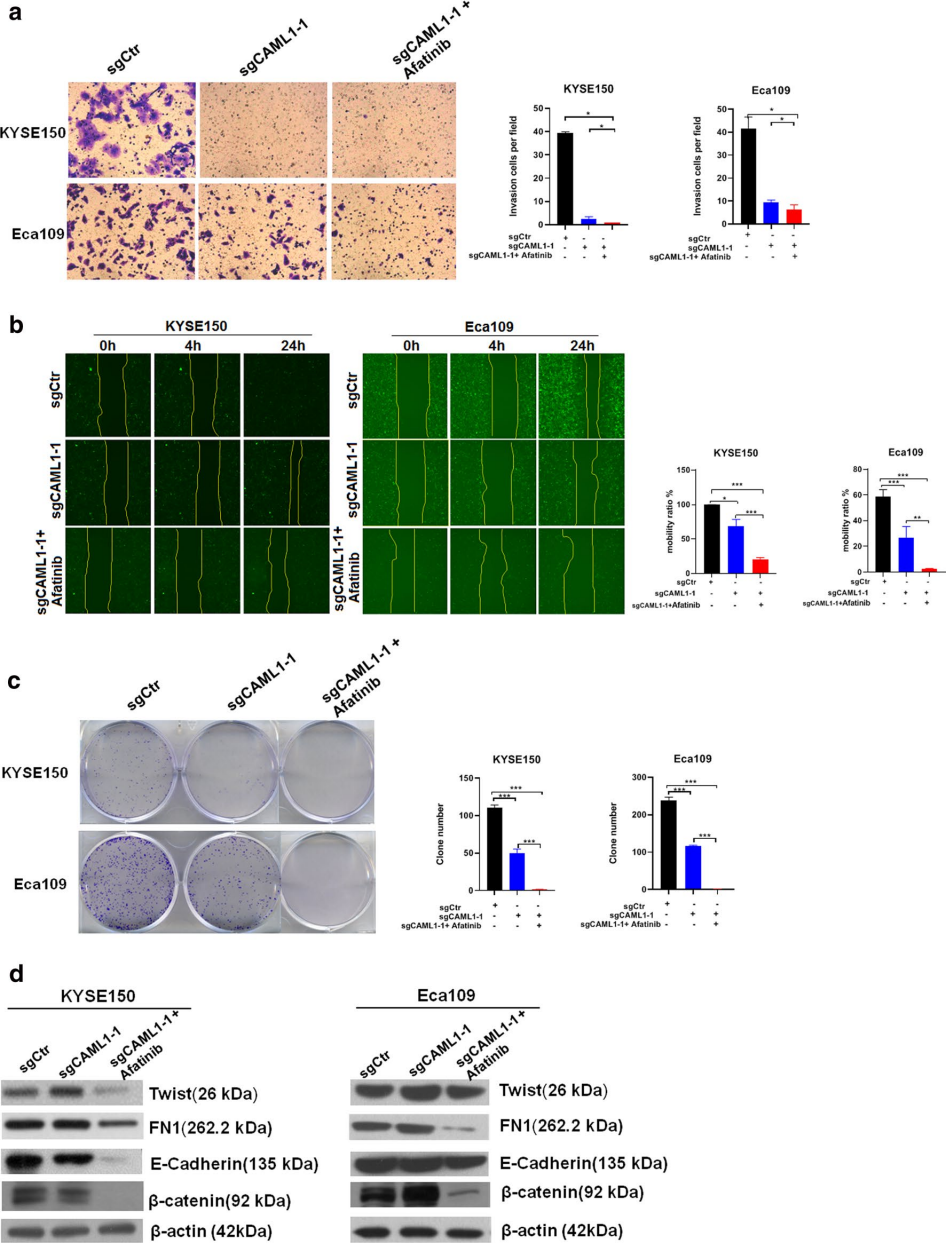
Knockout of CALM1 and application of EGFR inhibitor can synergistically inhibit the invasion and migration of ESCC. **a**, **b**, variation of invasive and migration ability was assessed by Transwell assay and Wound-healing assays in KYSE150 and Eca109 cell lines. **c** Colony-formation assay to quantify the combined effect of CALM1 and EGFR on ESCC viability. **d** Correspondingly, expression variation of biomarkers of ESCC cell lines KYSE150 and Eca109 related to EMT on protein level using immunoblotting. *CALM1* calmodulin1, *EGFR* epidermal growth factor receptor. *ESCC* esophageal squamous cell carcinoma, *EMT* epithelial-mesenchymal transition. *P < 0.05, **p < 0.01, ***p < 0.001 (Student's t-test)

**Fig. 4:**
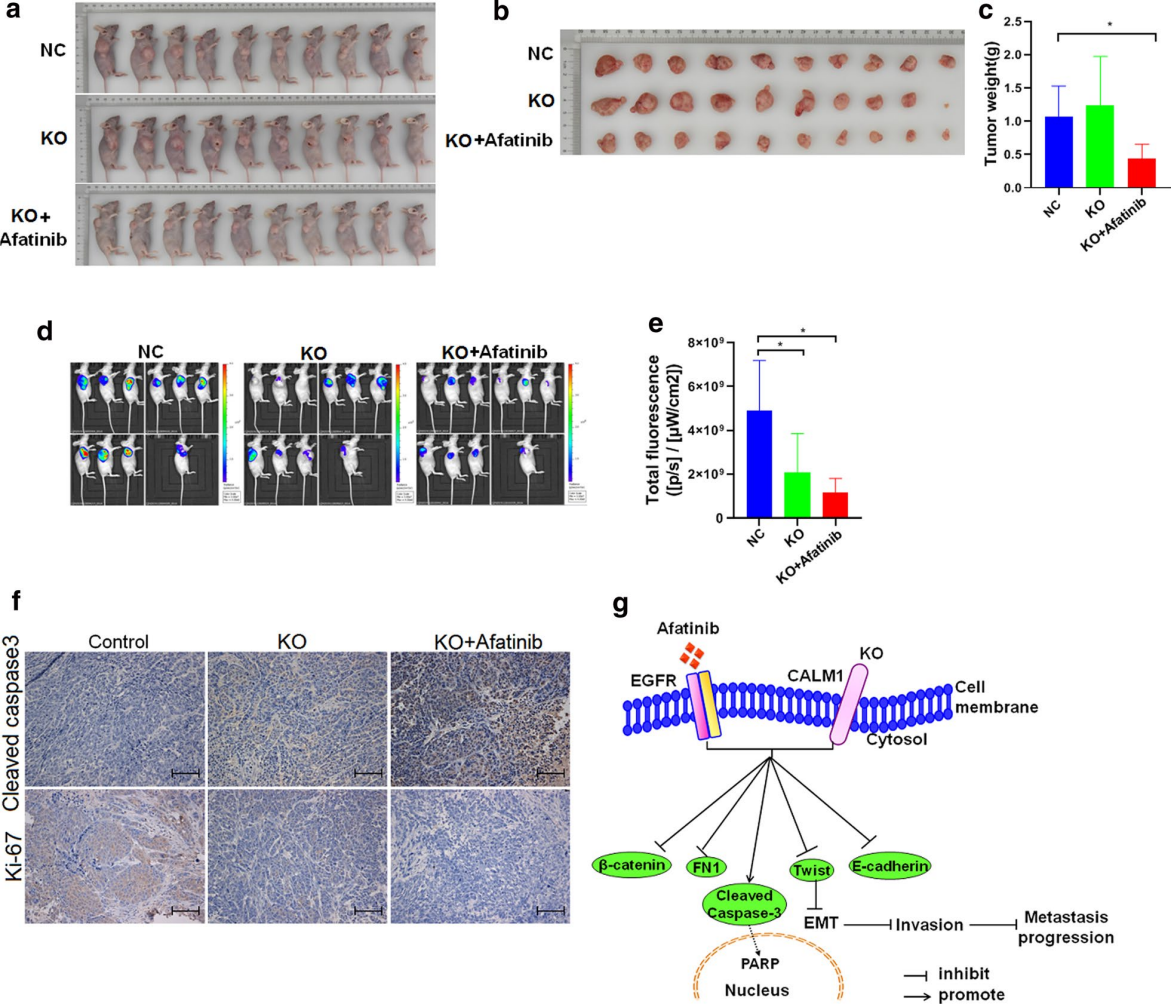
Effects of Knockout of CALM1 and application of EGFR inhibitor on tumorigenesis in nude mice in *vivo.* KYSE150 cells that were infected with CALM1 or scramble lentivirus were injected s.c. into nude mice. **a-c** Nude mice harboring subcutaneous tumors derived from implanted Control CALM1 and CALM1 KO cells were treated by subcutaneous injection with vehicle (saline) or Afatinib. Tumor dimensions were recorded on every other day and calculated tumor volumes are presented as the mean ± SD for each group (n = 10). Weight of terminal tumors was recorded on Day 27. **d, e **The total radiant efficiency of the ROI in xenografts from mice injected with Control CALM1 and CALM1 KO cells with vehicle (saline) or Afatinib. CALM1, calmodulin1; EGFR, epidermal growth factor receptor. ESCC, esophageal squamous cell carcinoma; *ROI* Region of Interest; *P < 0.05, **p < 0.01, ***p < 0.001 (Student's t-test). F, Immunostaining for Cleaved caspase-3 and Ki-67 on tumor lesions dissected from nude mice in the three groups. KO, knock-out of CALM1. Scale bar, 100 μm. Shown was the representative figures selected among candidates. G, Schematic diagram of the findings we described in our paper

### Knockout of CALM1 and treated with EGFR inhibitors markedly inhibited the invasion and migration of ESCC via EMT

Next, to investigate the combined effect of knock-out of CALM1 and EGFR inhibitor that exerted over proliferation of ESCC cells, we carried out Transwell assay and Wound-healing assays after KYSE150 and Eca109 were transfected with lentiviral-based knockout of CALM1. As shown in Fig. 3a, b, The results showed that compared with the control, the cell invasion and migration of ESCC cells were significantly suppressed in the sgCALM1-1 group compared with control group; however, a stronger increase was observed in the sgCALM1-1 group plus EGFR inhibitors group (p < 0.001). In clonogenic assay, we also found that silencing of CALM1 in combination with Afatinib caused a marked inhibition of proliferation in two cell lines, which is consistent with our previous results (p < 0.001, Fig. 3). We further focused on the mechanisms underlying CALM1 and EGFR activity in ESCC by examining the levels of FN1, the marker of EMT that was reported to be involved in cell invasion and migration. It turned out that no significant variation of FN1 expression can be observed after transfection with sgCALM1-1 compared with control group. By contrast, continued transfection with sgCALM1-1 with the addition of Afatinib, which led to the significant down-regulation of FN1 compared with control group (Fig. 3d). Much like FN1, β-catenin, another important player involved in migration and invasion of cancer cells, whose expression showed no change in group transfected with sgCALM1-1. While, it was remarkably reduced in group transfected with sgCALM1-1, followed by treatment with Afatinib. Besides, it should be noted that expression variation of E-cadherin was not evident in KYSE-150 cell line after treatment with Afatinib together with KO of CALM1; in stark contrast, the variation of E-Cadherin was significant in Eca109 cells. Collectively, our data suggest that CALM1 and EGFR contribute to tumor cell migration and invasion through promoting EMT.

### Knockout of CALM1 and treated with EGFR inhibitors markedly impaired the tumorigenesis in nude mice in vivo

To confirm the results of knockout of CALM1 and application of EGFR inhibitor in vivo mouse tumorigenesis model, where mice were injected with KYSE150 cells from the sgCtrl or sgCALM1-1 groups with vehicle (saline) or Afatinib, was generated. The results of tumor weight analysis revealed that sgCALM1-1 cells with Afatinib generated markedly smaller subcutaneous xenograft tumors in nude mice compared with sgCtrl and sgCALM1-1 cells group (Fig. 4a–c). In addition, in order to further confirm that knockout of CALM1 and treatment of EGFR inhibitor was directly associated with the observed effects on tumor growth, a fluorescence imaging test was also conducted using a small animal live imaging system, which monitors the fluorescence signals emitted from tissues. The sgCtrl-infected and sgCAML1-infected KYSE150 cells were also transduced with GFP; therefore, tumor xenografts in three groups emit fluorescence signals when triggered by specific fluorescence in the live imaging system in vivo. The fluorescence imaging results demonstrated that the total radiant efficiency of mice in the sgCAML1-infected group with treatment of EGFR inhibitor was markedly reduced compared with in the sgCtrl-infected group and sgCAML1 group (Fig. 4d–e). Moreover, to further confirm, tumor lesions dissected from nude mice in the three different groups we set were immunostained for cleaved caspase 3 and Ki-67, showing that expression of cleaved caspase 3 was higher in KO + Afatinib group, relative to controls; Contrary to cleaved caspase-3, staining of Ki-67 was lower in KO + Afatinib group compared with controls (Fig. 4f). These results clearly demonstrated that inhibition of CAML1 sensitized Afatinib treatment in vivo. Collectively, our data indicate that KO of CALM1 combined with EGFR inhibitor Afatinib displays synergistic effect in the suppression of metastasis of ESCC cells by suppressing the EMT process (Fig. 4g).

## Discussion

In the present investigation, we found that CALM1 and EGFR were remarkably up-regulated in ESCC, compared with paired NAT and that over-expression of CALM1 and EGFR in ESCC was significantly associated with tumor progression and poor overall prognosis. Furthermore, to functionally analyze the role of CALM1 in ESCC cell lines in vitro, KYSE150 and Eca109 cells were employed, whose endogenous CALM1 was down-regulated, respectively, by using lentiviral-based transfection. The combined effect of knock-out of CALM1 mediated by the lentivirus and EGFR inhibitors were shown to be capable of inhibiting the proliferation, cell cycle and increasing apoptosis of KYSE150 and Eca109 cells in vitro; invasion and migration were also depressed and enhancing epithelial-mesenchymal transition (EMT). In addition, to investigate the synergistic effect of CALM1 and EGFR plays in cell proliferation, nude mice were xenografted with ESCC cells whose CALM1 was stably knowdown in vivo.

While extensive research has shown that synergy between CALM and EGFR promotes gene transcription and cell proliferation in different cancer types, including human breast cancer, lung cancer, and astrocytic gliomas [10, 29, 30] but there are rare data in regard to its role in ESCC, especially CALM1. Kobayashi H et al. found that only CALM 1 played a role in the migration of mouse precerebellar neurons (PCNs) in vivo, while CALM2、CALM3 genes did not functionally replace CALM1. When the CALM1 is knocked down with the sgRNA, the radial and tangential migration of the cells is inhibited, and the final goal failded to reach during the development, but there is no harmful effect after knocking down the CALM2、CALM3 [31]. Huang et al. [32] obtained the first experimental evidence for CALM binding to the EGFR in a Ca^2+^-dependent manner in rat liver. In addition, the occurrence of CALM/EGFR complexes in living cells was established and the possible functional effects of this interaction on ligand-dependent activation were identified [33–35]. Based on these studies, it has firstly confirmed the expression of CALM1 and EGFR using IHC with ESCC tissue array. CALM1 and EGFR was upregulated in ESCC relative to NAT, and significantly correlated with poor overall prognosis, in the present study. By expanding the quantities of samples, further results were obtained showing that CALM1 and EGFR-positive staining is positively correlated with tumor progression and poor overall prognosis. Unlike CALM1 that has been seldom reported in the setting of ESCC, studies of EGFR in tumor are relatively extensive.

Anti-EGFR antibodies play an anti-tumor role by binding to cell surface receptors and interfering ligand binding, which leads to the inhibition of its downstream signaling pathway. Approved bare antibodies for EGFR (i.e.panituzumab, nimotuzumab, cetuximab, and necitumumab) have demonstrated their therapeutic efficacy in malignant tumors, but are usually used in combination with chemotherapy drugs to achieve significant clinical efficacy [36, 37]. Although the overexpression or mutation of EGFR levels has proven to be a valid predictor of treatment outcome, the response rates in selected patients remain chemoresistance or poor prognosis in squamous cell carcinomas as well as other malignancies [38–40]. Therefore, future research should focus on exploring more biomarkers to optimize the therapeutic effect on EGFR inhibitors. CALM inhibitors plays an essential role in cell proliferation and/or reverse multiple drug resistance tendencies in many tumor cells [41, 42], so it has been thought it has potential therapeutic effects in cancer [43, 44]. Based on many studies which mostly performed in vitro point to a potential benefit of treating cancers with CALM antagonists. A, V et al. [29] found that the site(s) of action of CALM in specific CALM-dependent systems that are upregulated in tumor cells interacting with EGFR. Ca^2+^-CALM binding to the CALM binding domain (CALM-BD) of cytosolic juxtamembrane region of the receptor plays an important trigger role in ligand-dependent activation EGFR in living cells [45, 46]. A further study was shown that non-phosphorylated CALM only interacts with the EGFR when is not phosphorylated at Tyr^1173^(tyrosine^1173^) [47]. Herein, We found here for the first time that treatment with knock-out of CALM1 and EGFR inhibitors have significant effects against tumors in vivo and in vitro in ESCC. Recent studies have pointed to the potential for combinations of EGFR inhibitors with TKIs to overcome a certain degree of resistance for EGFR mutations [48]. However, such strategies may be limited for special resistant population. In the current observation, knock-out of CALM1 mediated by the lentivirus turns out to be able to slow down the growth and motility of KYSE150 and Eca109 cells, preliminarily defining the oncogenic roles of CALM1 in ESCC cells. Further, our combined use of EGFR inhibitors significantly reduced cell proliferation, invasion and migration in vitro and in vivo*.* Our study provided a molecular phenotype for ESCC, suggesting that CALM1 and EGFR inhibitors might be used as a potential therapeutic target for patients with ESCC. Despite this, there were still some limitations we acknowledged including, the number of clinical samples, totaling 50, was not big enough, which was one of its inborn limitations. In addition, the number of ESCC cell lines involved was also limited. Last but not least, we explored the expression status as well as the biological roles of EGFR and CALM1 in ESCC tissues and cell lines; though, we failed to understand the mutational status of EGFR in ESCC. In addition, a large number of clinical studies revealed that there were some mutation points of EGFR in ESCC, in our study; we did not explore the mutation status. Given these, in the future investigation, the working mechanism by which CALM1 works and mutation status of EGFR in ESCC should be carried out to better understand how CALM1 and EGFR function in ESCC and further study is warranted.

In conclusion, the combined effect of CALM1 and EGFR was observed to be able to remarkably inhibit tumor development in KYSE150 and Eca109 cells, suggesting that the combined effect of CALM1 and EGFR may assist in the development of new therapeutic strategies to enhance treatment efficacy of EGFR-targeted therapy.

## Supplementary Information

**Additional file 1: Fig. S1.**The IC50 value of Afatinib for KYSE150 and Eca109 cells were shown, respectively. The IC50, half-maximum inhibitory concentration.

## Data Availability

Source data and materials can be available from the corresponding author on reasonable request.
