# Supplementary figures and images for: CALM1 promotes progression and dampens chemosensitivity to EGFR inhibitor in esophageal squamous cell carcinoma
Source: Cancer Cell Int. 2021 Feb 18;21:121. doi: 10.1186/s12935-021-01801-6 (PMC7890995; doi:10.1186/s12935-021-01801-6)

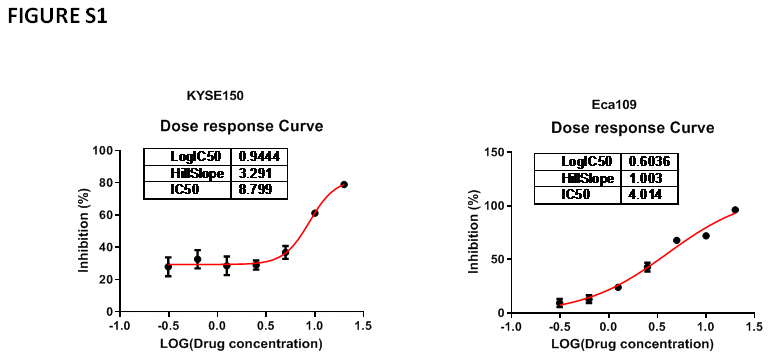

Supplement: Supplementary file 1 — Additional file 1: Fig. S1.The IC50 value of Afatinib for KYSE150 and Eca109 cells were shown, respectively. The IC50, half-maximum inhibitory concentration. [file 12935_2021_1801_MOESM1_ESM.tif]
